# Influence of Catheter–Incision Congruency in Epidural Analgesia on Postcesarean Pain Management: A Single-Blinded Randomized Controlled Trial

**DOI:** 10.3390/jpm11111099

**Published:** 2021-10-27

**Authors:** Ying-Hsi Chen, Wei-Han Chou, Jr-Chi Yie, Hsiao-Chun Teng, Yi-Luen Wu, Chun-Yu Wu

**Affiliations:** 1 Department of Anesthesiology, National Taiwan University Hospital, Taipei 100, Taiwan; yhc2248@gmail.com (Y.-H.C.); brokenarrowchou@yahoo.com.tw (W.-H.C.); yiejrchi@gmail.com (J.-C.Y.); laurieteng@gmail.com (H.-C.T.); 2 Department of Medical Education, National Taiwan University, Taipei 100, Taiwan; b03401126@ntu.edu.tw

**Keywords:** postcesarean pain, epidural congruency, patient-controlled epidural analgesia, epidural morphine

## Abstract

Patient-controlled epidural analgesia (PCEA) or epidural morphine may alleviate postcesarean pain; however, conventional lumbar epidural insertion is catheter–incision incongruent for cesarean delivery. Methods: In total, 189 women who underwent cesarean delivery were randomly divided into four groups (low thoracic PCEA, lumbar PCEA, low thoracic morphine, and lumbar morphine groups) for postcesarean pain management. Pain intensities, including static pain, dynamic pain, and uterine cramp, were measured using a 100 mm visual analog scale (VAS). The proportion of participants who experienced dynamic wound pain with a VAS score of >33 mm was evaluated as the primary outcome. Adverse effects, including lower extremity blockade, pruritus, postoperative nausea and vomiting, sedation, and time of first passage of flatulence, were evaluated. Results: The low thoracic PCEA group had the lowest proportion of participants reporting dynamic pain at 6 h after spinal anesthesia (low thoracic PCEA, 28.8%; lumbar PCEA, 69.4%; low thoracic morphine, 67.3%; lumbar morphine group, 73.9%; *p* < 0.001). The aforementioned group also reported the most favorable VAS scores for static, dynamic, and uterine cramp pain during the first 24 h after surgery. Adverse effect profiles were similar among the four groups, but a higher proportion of participants in the lumbar PCEA group (approximately 20% more than in the other three groups) reported prolonged postoperative lower extremity motor blockade (*p* = 0.005). In addition, the first passage of flatulence after surgery reported by the low thoracic PCEA group was approximately 8 h earlier than that of the two morphine groups (*p* < 0.001). Conclusions: Epidural congruency is essential to PCEA for postcesarean pain. Low thoracic PCEA achieves favorable analgesic effects and may promote postoperative gastrointestinal recovery without additional adverse effects.

## 1. Introduction

Postcesarean pain has been ranked ninth for pain severity among 179 different surgical procedures in the first 24 h after surgery [1], and women often claim that they received inadequate analgesia after cesarean delivery [2]. Inadequate postcesarean pain management is associated with multiple negative maternal effects such as delayed postpartum recovery [3,4], interference with breastfeeding [5], and a high risk of postpartum depression and persistent pain [6]. Epidural analgesia achieves better analgesic effects than do parenteral opioids in the surgical population [7], and combined spinal-epidural anesthesia is regarded as a suitable option for cesarean delivery [8,9]. Therefore, epidural analgesia may play a key role in optimizing postcesarean pain management; however, the related mechanism has not been sufficiently explored.

At least two common epidural modalities may be applied for postcesarean analgesia, namely patient-controlled epidural analgesia (PCEA) with local anesthetic-based drugs and epidural morphine. However, the analgesic efficacy of PCEA versus epidural morphine for cesarean delivery remains inconclusive partly because the role of the epidural insertion site has never been examined. Epidural catheter–incision congruency is achieved when the placement of the epidural catheter corresponds to the dermatomes of the surgical incision, and it substantially influences epidural local anesthetic efficacy [10]. In this regard, a low thoracic epidural insertion has greater epidural catheter–incision congruency related to postcesarean pain than has conventionally recommended lumbar insertion [11,12,13]. However, the influence of epidural catheter–incision congruency with respect to PCEA and epidural morphine has never been prospectively investigated. Therefore, we conducted a randomized controlled trial to compare the profiles of postcesarean pain and epidural-related adverse effects related to two epidural insertion sites (i.e., the low thoracic and lumbar regions) in women undergoing elective cesarean delivery.

## 2. Materials and Methods

### 2.1. Participants and Preoperative Evaluation

This is a single-blinded randomized controlled trial of a single center. Ethical approval for this study (201902009RINC) was granted by the Research Ethics Committee of National Taiwan University Hospital, Taipei, Taiwan. This study was registered at ClinicalTrials.gov (identifier: NCT03946982). We enrolled adult women who underwent elective cesarean delivery between 8 a.m. and 12 p.m. on weekdays between June 2019 and April 2021. Study candidates were excluded if they met the following exclusion criteria: contraindication for epidural analgesia such as coagulopathy or refusal, preeclampsia status, or having received perioperative magnesium infusion (because magnesium may reduce analgesic consumption) [14].

An investigator who was independent of clinical care obtained written informed consent from each participant on the day before surgery. Additionally, a three-item preoperative screening questionnaire was administered. Designed to predict postcesarean pain, the questionnaire assessed preoperative anxiety levels (“On a scale of 0–100, with 0 being not anxious at all through 100 being extremely anxious, how anxious are you about your upcoming surgery?”), anticipated postcesarean pain (“On a scale of 0–100, with 0 being no pain at all and 100 being the worse pain imaginable, how much pain do you anticipate experiencing after your upcoming surgery?”), and rating of anticipated pain medication needs (“On a scale of 0–5, with 0 being none at all, 1 being much less than average, 2 being less than average, 3 being average, 4 being more than average, and 5 being much more than average, how much pain medication do you anticipate needing after your upcoming surgery?”) [15]. Upon arriving at the operating room, participants were allocated to the study arms based on a predefined block randomization list. Accordingly, the participants were randomly divided at an equal ratio into four groups, namely the low thoracic PCEA, lumbar PCEA, low thoracic morphine, and lumbar morphine groups. 

### 2.2. Epidural Catheterization and Spinal Anesthesia

Regional anesthesia for each participant was administered in the lateral decubitus position by employing the separate space technique [16]. The intervertebral spaces for epidural and spinal insertion were identified using ultrasound; this technique was similar to that used in a previous study [17]. The interlaminar space between L5 and the sacrum was first identified, and the operator then counted up to locate the planned insertion site for spinal anesthesia and epidural analgesia. The epidural insertion levels were T11–12 and L3–4 for the low thoracic and lumbar groups, respectively. The epidural multiport catheter was inserted using a 16-gauge Tuohy needle (Portex, Smiths Medical ASD, Keene, NH, USA) and threaded 5 cm into the epidural space. During epidural insertion, an independent investigator recorded the procedure time and the number of additional attempts made to perform the insertion. An additional attempt was defined as one in which the needle had to be withdrawn for redirection or reinsertion. 

After the epidural catheter was placed, spinal anesthesia was administered to each participant at L4–5 by using a 27-gauge BD Quincke spinal needle (Becton Dickinson, Madrid, Spain) and a combination of 12 mg of 0.5% hyperbaric bupivacaine and 15 µg of fentanyl to achieve a T6 sensory blockade [18,19]. Attending anesthesiologists could administer epidural analgesic with 5–10 mL of 2% lidocaine without epinephrine per dose for inadequate intraoperative analgesia; however, long-acting agents such as bupivacaine and morphine were not allowed for epidural administration to avoid bias in postcesarean pain assessments.

The PCEA regimen comprised 0.66 mg/mL bupivacaine and 1.75 µg/mL fentanyl in the initial setting; it was designed for the delivery of a program-intermittent bolus infusion of 3 mL and demand dose of 4 mL at a lockout interval of 20 min and with a 4 h limit of 40 mL. PCEA was initiated with the first bolus infusion being performed at 1 h after surgery. Participants in the epidural morphine group received 2 mg of epidural morphine (which was mixed with sterile saline to a volume of 10 mL) at the end of surgery and at 9 a.m. and 9 p.m. on the 2 days that followed. Furthermore, at the end of the surgery, 4 mg of ondansetron was intravenously administered to each participant for prophylaxis of nausea, vomiting, and pruritus.

### 2.3. Primary Outcome: Pain Intensity Assessment

Postcesarean pain intensity was assessed by investigators who were independent of the clinical care team; the investigators used a 100 mm visual analog scale (VAS) [20,21,22] to assess the three categories of pain, namely static pain (wound pain at rest), dynamic pain (wound pain during movement or activities), and uterine cramp pain. The VAS for the three pain categories was assessed at four time points, that is, at 6 h after spinal anesthesia (T_1_), between 9 a.m. and 10 a.m. on postoperative day 1 (T_2_); between 3 p.m. and 5 p.m. on postoperative day 1 (T_3_), and between 9 a.m. and 10 a.m. on postoperative day 2 (T_4_). During each assessment, the investigator and participant were blinded to the previous VAS questionnaire results. Based on a recent analysis of objective acute postoperative pain assessments that used the 100-mm VAS, a VAS position marking of ≤33 mm indicates an acceptable postoperative pain control response [22]. We expected the most intense postcesarean pain to occur after the resolution of spinal anesthesia [23] and that dynamic pain would be a major source of postoperative pain [20]. Accordingly, the primary outcome of this study was the proportion of unacceptable pain control in a group, with a nonresponse being defined as a dynamic pain VAS score of >33 mm at T_1_.

### 2.4. Rescue and Adjuvant Analgesics

During the 2 days following surgery, oral analgesics were provided, including 500 mg of acetaminophen that was administered every 6 h and 500 mg of naproxen [24] that was administered twice daily. The participants were instructed to ask for rescue analgesia if they experienced inadequate pain relief; this measure was independent of the VAS assessment results. Postpartum ward personnel used a rescue analgesia protocol that comprised 10 mg of nalbuphine pro re nata (PRN) that was intravenously administered every 6 h for breakthrough pain and 40 mg of tenoxicam PRN that was intravenously administered every 12 h for inadequately controlled uterine cramping. When postcesarean pain remained inadequately controlled after the initial intravenous administration of rescue analgesic at the postpartum ward, the acute pain service team was then allowed to adjust the programmed intermittent bolus dose for the participants in the two PCEA groups. When acute pain management service was inadequate during night shifts, 1 mg of supplementary epidural morphine was administered to participants in the four study groups who were subjectively unsatisfied with their analgesia quality after undergoing the rescue analgesia protocol at 7 p.m.

### 2.5. Secondary Outcome: Adverse Effects of Epidural Analgesia

The adverse effects of epidural analgesia include lower extremity blockade, pruritus, postoperative nausea and vomiting, and severe sedation; the evaluation of these effects was conducted concurrently with the primary outcome assessment. The adverse effects were compared with the proportion of participants in a group who experienced the most severe effects during the investigation period. Lower extremity blockade was assessed using a modified Bromage motor scale, which is a 4-point scale in which a score of 0 indicates an individual is able to lift his or her legs against gravity (i.e., no motor blockade), 1 indicates an individual is able to flex his or her knees but not his or her legs, 2 indicates an individual is able to move his or her feet without being able to flex his or her knees, and 3 indicates an inability to move any joints (i.e., full paralysis) [25]. The degree of pruritus was categorized as 0 (no pruritus or mild pruritus with no request for treatment), 1 (moderate pruritus with the request for a single type of antipruritic drug), or 2 (severe pruritus with the request for multiple types of antipruritic drugs). Pruritus was firstly treated using oral antihistamine medication (levocetirizine) that was administered PRN every 8 h. For severe pruritus, antihistamine medication or ondansetron may be intravenously administered at the discretion of the attending clinician [26]. Nausea was defined as any unpleasant sensation with awareness of the urge to vomit. Vomiting was defined as the successful or unsuccessful (retching) expulsion of gastric contents [27] and treated by the intravenous administration of 4 mg of ondansetron PRN every 8 h. The severity of opioid-induced sedation was assessed using the Ramsay Sedation Scale (1, anxious patient; 2, cooperative and tranquil; 3, responsive to commands; 4, brisk response to stimulus; 5, sluggish response to stimulus; 6, no response to stimulus) [28]. The number of participants presenting with a Ramsay Sedation Scale score that was not 2 was evaluated. Furthermore, the time of first passage of flatulence after surgery reported by the four groups was recorded and compared. Recovery questionnaires such as the ObsQoR-11 have been reported to produce reliable assessments of postcesarean recovery [29], but such questionnaires have not yet been validated in our language. Therefore, the postcesarean recovery was assessed by rating the global health with a numerical rating scale of 0–100 at 48 h after surgery. A numeric rating scale ≥ 70 was considered as a good recovery which was used as a discriminant validity in the development of postoperative quality of recovery scoring systems for both surgical and obstetric populations [29,30].

### 2.6. Statistical Analysis

The incidence of moderate-to-severe postcesarean pain (unacceptable pain control) is 50%–70% [15,31]. Accordingly, to obtain an at least 15% reduction in unacceptable postcesarean pain than the lower limit in the literature (50%), a minimal sample of 176 participants, divided into four groups (each comprising of 44 participants), were analyzed to differentiate the 35%, 50%, 50%, and 70% of participants among the four groups who reported a dynamic VAS score of >33 mm at T_1_ with a power of 0.8 and at a significance level of 0.05. Given the risk of epidural failure and frequent clinical labor force requirements for emergent cesarean procedures (because our institute is a tertiary obstetric referral center), 200 participants (50 in each group) were enrolled. The proportional data were compared using chi-squared tests followed by the Marascuilo procedure when appropriate. One-way analysis of variance and Tukey’s test were performed to compare the means of the four groups; repeated-measures analysis of variance (with group and time factors) and Tukey’s test were performed to compare the VAS trajectories of the four groups from T_1_ to T_4_. 

To compare analgesic consumption between two groups (low thoracic PCEA vs. lumbar PCEA; low thoracic morphine vs. lumbar morphine), Student’s *t*-test was performed for normally distributed continuous data, and the Mann–Whitney *U* test was performed for nonparametric ordinal data. Statistical analyses were performed using PASS 2021 Sample Size Software (NCSS, Kaysville, UT, USA) and MedCalc Statistical Software version 20 (MedCalc, Ostend, Belgium).

## 3. Results

**Figure 1:**
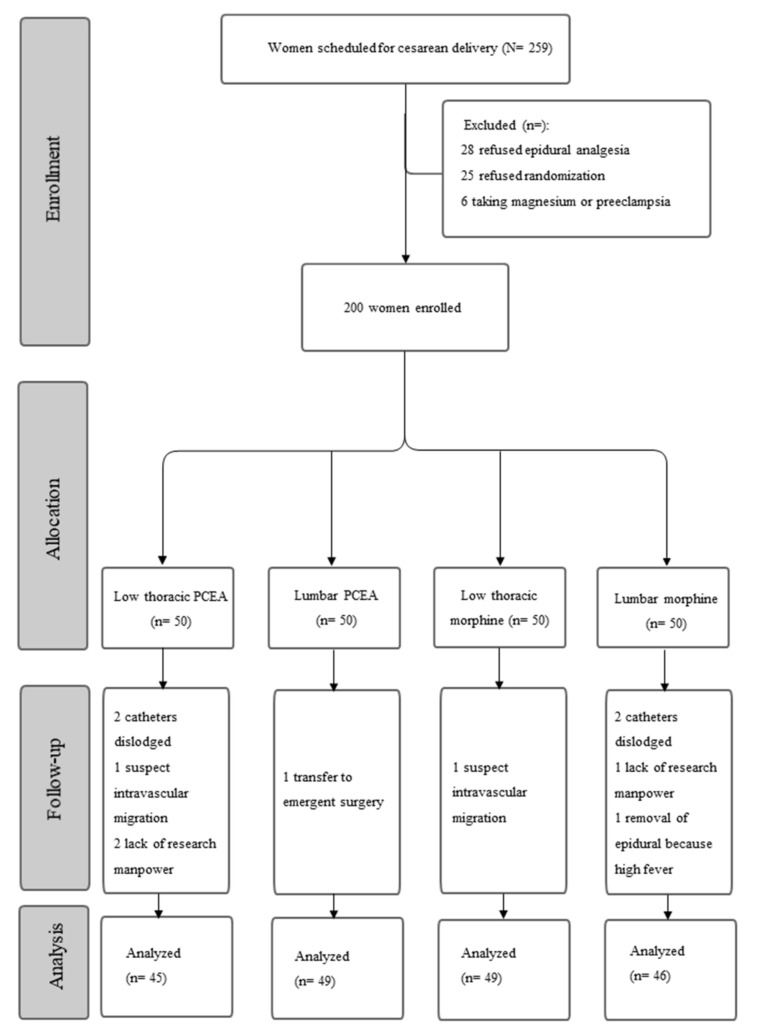
CONSORT flowchart.

Figure 1 presents the participant inclusion and exclusion flowchart. In total, 189 participants were enrolled for the final analysis, which included 45 participants in the low thoracic PCEA group, 49 participants in the lumbar PCEA group, 49 participants in the low thoracic morphine group, and 46 participants in the lumbar morphine groups, respectively. There were 11 participants excluded during the follow-up. The patients were excluded due to seven failed epidurals (four participants with a dislodged epidural catheter; two participants with suspected intravascular migration of catheter and one participant with early removal of the epidural catheter due to high fever), and three participants with a lack of research manpower, and one participant was transferred to emergent delivery due to unstable fetal heartbeats. 

Demographic characteristics and preoperative obstetric data (i.e., age, weight, height, parity, and indication for cesarean delivery) and preoperative questionnaires were compared among the four groups (Table 1).

### 3.1. Intraoperative Profiles

Table 2 summarizes the intraoperative profiles of the four groups. Operation time and blood loss were similar among the four groups. 

Furthermore, no significant difference among the four groups was observed for skin-epidural depth, time for epidural catheterization, and the number of epidural redirection attempts. Compared with the two lumber groups, those in the two low thoracic groups had a nonsignificantly higher tendency to exhibit higher spinal anesthesia sensory blockade levels (*p* = 0.054); however, the requirements for intravenous fluid and norepinephrine were similar among the four groups. 

### 3.2. Postcesarean Pain: Proportion of Participants with a VAS Score of >33 mm

**Figure 2:**
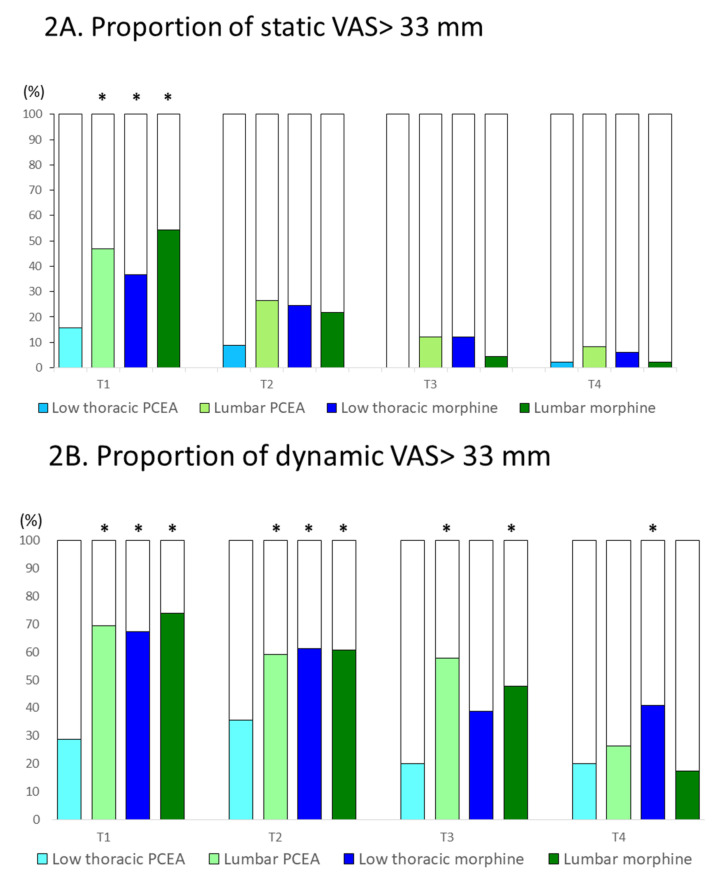

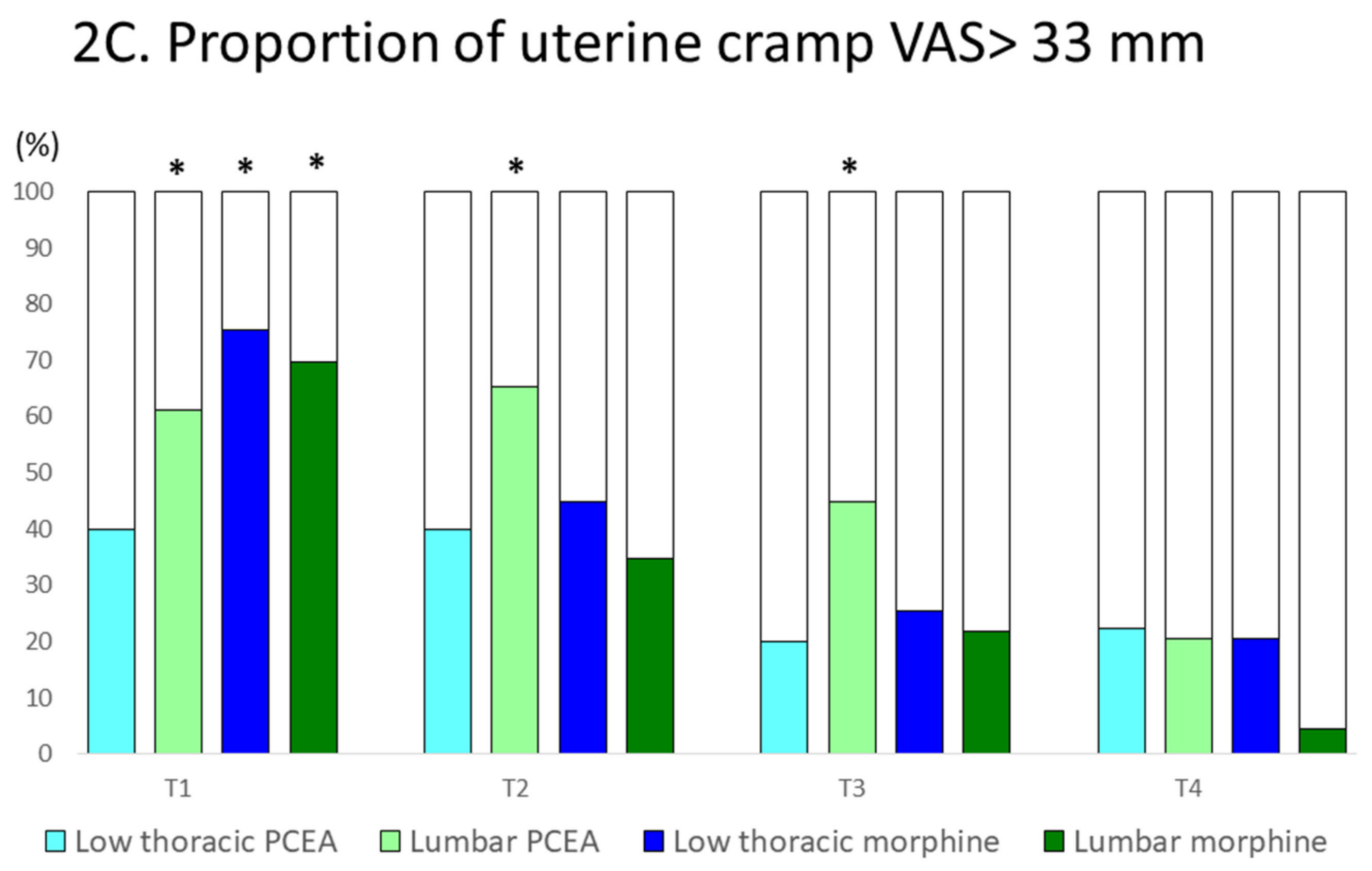
(**A**) Proportion of participants with a visual analog scale score of >33 mm of static wound pain. * means a *p* value < 0.05 compared to that of the low thoracic PCEA group. (**B**) Proportion of participants with a visual analog scale score of >33 mm of dynamic wound pain. * means a *p* value < 0.05 compared to that of the low thoracic PCEA group. (**C**) Proportion of participants with a visual analog scale score of >33 mm of uterine cramp. * means a *p* value < 0.05 compared to that of the low thoracic PCEA group.

The low thoracic PCEA group had the lowest proportion of participants reporting a VAS score of >33 mm (Figure 2). Detailed information of the proportions of participants among the four study groups with a VAS of >33 mm was provided in the Supplementary Materials (Table S1). Among the four study groups, the highest proportions of a VAS score of >33 mm in the three categories of pain (static pain, dynamic pain, and uterine cramp) was presented at T_1_ (Figure 2A–C) except for the lumbar PCEA group, which had more participants presenting with a uterine cramp of VAS > 33 mm at T_2_ (Figure 2C). Accordingly, we observed significant analgesic effects of low thoracic PCEA in the prevention of unacceptable postcesarean pain at T_1_. For instance, low thoracic PCEA achieved approximately 20–40% reduction in the proportion of participants with a static VAS of >33 mm at T_1_ (15.6%, 46.9%, 36.7%, and 54.3% of the participants in the low thoracic PCEA, lumbar PCEA, low thoracic morphine, and lumbar morphine groups, respectively, reported a static VAS score of >33 mm; *p* < 0.001; Figure 2A). 

The low thoracic PCEA had the most prominent effects in the prevention of postcesarean dynamic VAS score of >33 mm with approximately 40–45% reduction compared to the other three study groups at T_1_ (28.8%, 69.4%, 67.3%, and 73.9% of the participants in the low thoracic PCEA, lumbar PCEA, low thoracic morphine, and lumbar morphine groups, respectively, reported a dynamic VAS score of >33 mm; *p* < 0.001; Figure 2B). In addition, low thoracic PCEA also achieved approximately 20–35% reduction in the proportion of participants with a uterine cramp VAS of >33 mm at T_1_ (40. 8%, 61.2%, 75.5%, and 69.6% of the participants in the low thoracic PCEA, lumbar PCEA, low thoracic morphine, and lumbar morphine groups, respectively, reported a uterine cramp VAS score of >33 mm; *p* < 0.001; Figure 2C). 

Furthermore, the low thoracic PCEA group continued to report superior analgesic effects on postoperative day 1 and day 2 in the prevention of the dynamic VAS score of >33 mm and the uterine cramp VAS score >33 mm (Figure 2B,C).

### 3.3. Postcesarean Pain: Comparison of VAS Scores

**Figure 3:**
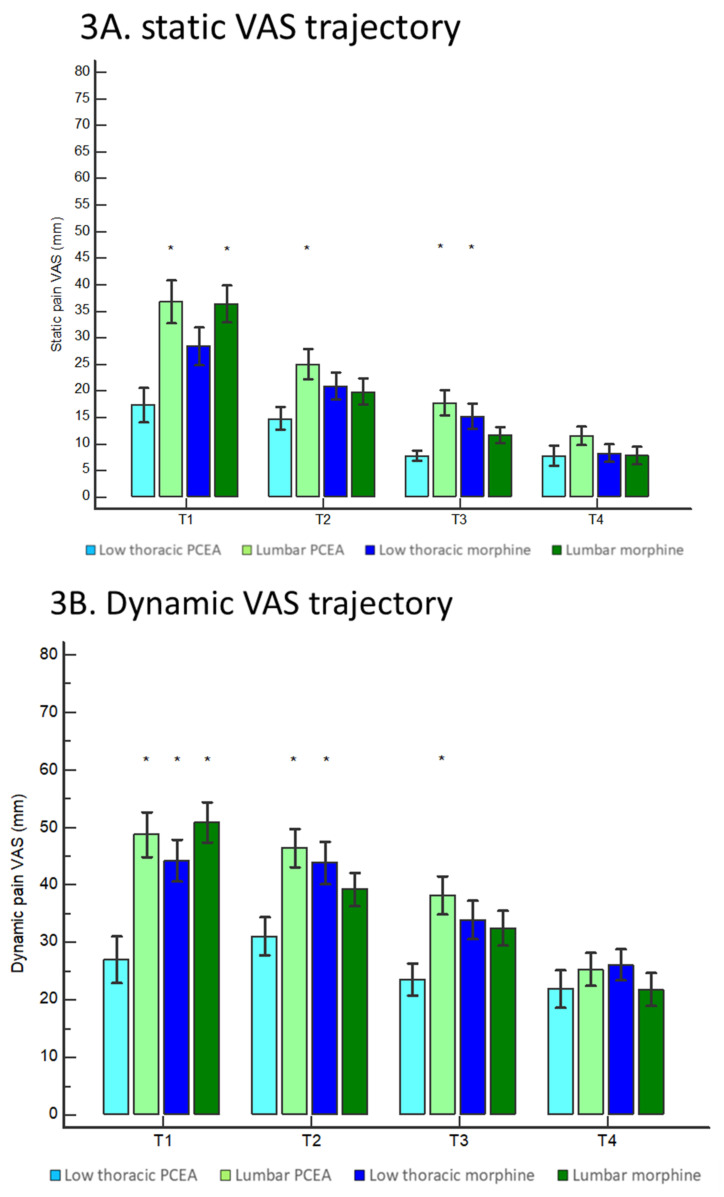

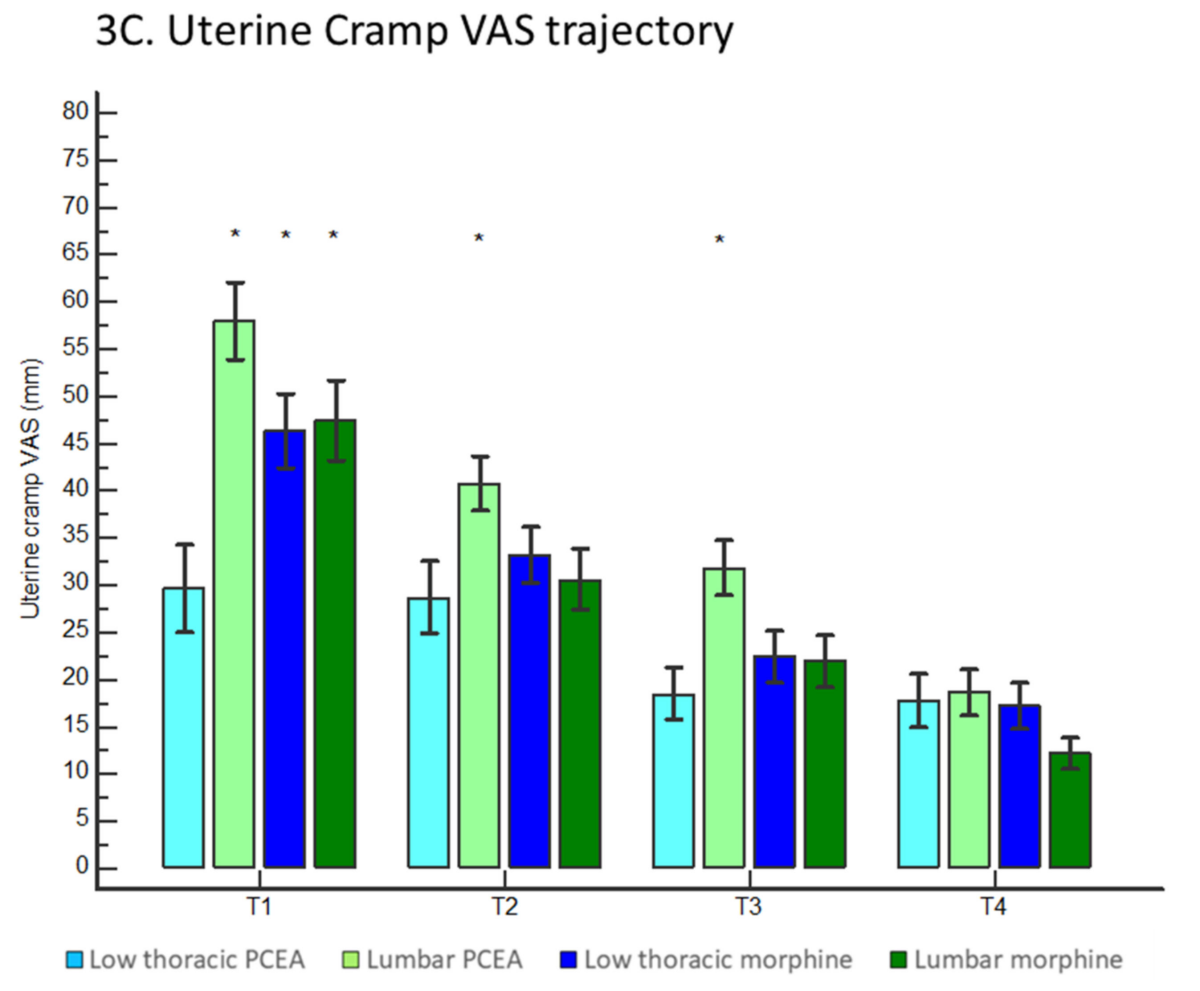
(**A**). Time trajectory changes in the visual analog scale of static wound pain. * means a *p* value < 0.05 compared to that of the low thoracic PCEA group. The low thoracic PCEA achieved a particularly superior static VAS score than those of the lumbar PCEA through T_1_ to T_3_. (**B**) Time trajectory changes in the visual analog scale of dynamic wound pain. * means a *p* value < 0.05 compared to that of the low thoracic PCEA group. The low thoracic PCEA achieved the lowest dynamic VAS score at T_1_ compared to the other three study groups. The beneficial analgesic effects of low thoracic PCEA were also observed at T_2_ and T_3_ when compared to those of the lumbar PCEA. (**C**). Time trajectory changes in the visual analog scale of uterine cramp. * means a *p* value < 0.05 compared to that of the low thoracic PCEA group. The low thoracic PCEA achieved the lowest uterine cramp VAS score at T_1_ compared to the other three study groups. The beneficial analgesic effects of low thoracic PCEA were also observed at T_2_ and T_3_ in comparison to those of the lumbar PCEA.

Similar to the reduction in the proportion of participants with a VAS of >33 mm, the beneficial analgesic effects of low thoracic PCEA on VAS scores were best observed at T_1_. For instance, the participants in the low thoracic PCEA group reported a median static VAS score of 9 (0–24) at T_1_, which was significantly lower than the VAS scores of 32 (1–56) and 38 (14–53) reported by the lumbar PCEA and lumbar morphine groups, respectively (Figure 3A; *p* < 0.001). The beneficial analgesic effects of low thoracic PCEA on static VAS score over lumbar PCEA were observed throughout T_1_ to T_3_ (Figure 3A).

At T_1_, the participants in the low thoracic PCEA group also reported a mean dynamic VAS score of 27 ± 27, which was significantly lower than the VAS scores of 49 ± 27, 44 ± 26, and 51 ± 24 reported by the lumbar PCEA, low thoracic morphine, and lumbar morphine groups, respectively (Figure 3B; *p* < 0.001). The beneficial analgesic effects of low thoracic PCEA on dynamic VAS score over lumbar PCEA were also observed at T_2_ and T_3_ (Figure 3B).

At T_1_, the participants in the low thoracic PCEA group reported a median uterine cramp VAS score of 15 (1–59), which was significantly lower than the VAS score of 61 (37–82), 45 (23–68), and 48 (24–72) reported by the lumbar PCEA, low thoracic morphine, and lumbar morphine groups (Figure 3C; *p* < 0.001). The beneficial analgesic effects on uterine cramp VAS score of low thoracic PCEA over lumbar PCEA were also observed at T_2_ and T_3_ (Figure 3C).

### 3.4. Adverse Effect Profiles

Table 3 summarizes the adverse effect profiles of the four epidural analgesia groups.

The detected Bromage motor scale scores of ≥1 were all reported at T_1_. No detectable increase in Bromage motor scale scores was observed throughout the remaining study period (i.e., T_2_ to T_4_) in the four groups. Compared with the other groups, a lower proportion of participants in the lumbar PCEA group scored 0 on the Bromage motor scale (low thoracic PCEA group, 91.1%; lumbar PCEA group, 69.4%; low thoracic morphine group, 87.7%; lumbar morphine group, 91.3%; *p* = 0.005). The incidence and severity of pruritus and postoperative nausea and vomiting were similar among the four groups (Table 2). Additionally, every participant in the four groups was cooperative and oriented (Ramsay sedation scale = 2) during the investigation period. However, the participants in the two epidural morphine groups reported a prolonged (approximately 6–8 h) first passage of flatulence after surgery relative to the participants in the two PCEA groups (low thoracic PCEA group, 17.3 ± 7.5 h; lumbar PCEA group, 20.8 ± 8.4 h; low thoracic morphine group, 26.2 ± 7.9 h; lumbar morphine group, 25.1 ± 7.6 h; *p* < 0.001). Furthermore, participants in the lumbar PCEA group revealed the worst recovery scores, and participants among the other three groups reported comparable scores (Table 2).

### 3.5. Analgesic Requirement

The participants in the low thoracic PCEA group required significantly lower doses of PCEA (lower by approximately 30%) compared with those in the lumbar PCEA group (low thoracic PCEA group, 206 mL (193–228 mL); lumbar PCEA group, 275 mL (228–308) mL, respectively; *p* < 0.001; Table 4). 

Compared with the participants in the lumbar PCEA group, those in the low thoracic PCEA group requested significantly fewer doses with respect to patient-controlled demand (low thoracic PCEA group, 9 (4–25); lumbar PCEA group, 21 (9–37); *p* = 0.007) and delivery (low thoracic PCEA group, 7 (2–18); lumbar PCEA group, 11 (7–25); *p* = 0.002) doses. The low thoracic PCEA group had a comparable demand/delivery ratio to the lumbar PCEA group (*p* = 0.371; Table 4). By contrast, the two epidural morphine groups reported similar levels of total epidural morphine consumption. 

In terms of rescue analgesic profile, the participants in the low thoracic PCEA and lumbar PCEA groups required, respectively, the fewest and most numbers of rescue analgesia intervention (low thoracic PCEA group, 1.0 ± 1.1; lumbar PCEA group, 2.5 ± 1.5; low thoracic morphine group, 1.6 ± 1.8; lumbar morphine group, 1.7 ± 1.7; *p* < 0.001; Table 3). Furthermore, participants in the low thoracic PCEA group also requested the lowest dose of supplement epidural morphine (low thoracic PCEA group, 0.1 ± 0.4 mg; lumbar PCEA group, 0.7 ± 0.9 mg; low thoracic morphine group, 0.9 ± 1.1 mg; lumbar morphine group, 0.8 ± 1.0 mg; *p* < 0.001; Table 4). The four groups had similar levels of nalbuphine and tenoxicam consumption.

## 4. Discussion

The findings of the present study verified the essential role of catheter–incision congruency in the application of PCEA for postcesarean pain. First, low thoracic PCEA provides better analgesic effects than lumbar PCEA and epidural morphine. Second, low thoracic PCEA is associated with a more favorable lower limb motor scale profile relative to lumbar PCEA and a faster first passage of flatulence compared with epidural morphine. Third, the difficulty of epidural catheter placement was similar for low thoracic and lumbar insertions.

The efficacy of low thoracic PCEA for postcesarean pain relief had not been prospectively investigated prior to the present study. The incidence of moderate-or-severe postcesarean pain at 24 h after surgery is between 50% and 78.4% [15,31], and the highest reported VAS score is between 40 and 85 mm for various analgesia methods, excluding low thoracic PCEA [15,23,32,33,34,35,36]. Furthermore, postpartum women are often reluctant to receive analgesics for fear of exposing their children to pain medication [2,37]. A previous study indicated that postpartum women could tolerate a pain level of 5.6 (standard deviation 2.2) despite being undertreated for postcesarean pain [37]. Therefore, instead of directly comparing the VAS scores of the four groups, the primary endpoint of the present study was the proportion of participants whose reported VAS scores exceeded the objective VAS score threshold of >33 mm. Nevertheless, we observed that relative to the other groups, the low thoracic PCEA group not only had a lower proportion of patients reporting a VAS of >33 mm; but also, these participants achieved a more favorable VAS score than did the other groups. In the present study, intravenous nalbuphine and tenoxicam were used institutionally because nalbuphine provides superior or comparable analgesic effects [36] to other opioids (such as sufentanil and morphine) but with fewer adverse effects [38]. In addition, intravenously administered nalbuphine efficiently ameliorates neuraxial opioid-induced pruritus without attenuating epidural analgesic effects [39]. Furthermore, tenoxicam has a long plasma half-life and can thus provide a longer period of relief for uterine cramp pain compared with other nonsteroid anti-inflammatory drugs [40]. The doses of rescue intravenous nalbuphine and tenoxicam were comparable between the four study groups. This may be because the multimodal analgesia was applied to the neuraxial analgesia, and thus the effect of specific rescue analgesic could be diluted. However, the participants in the low thoracic PCEA group required the fewest rescue analgesia interventions and supplementary epidural morphine. These findings highlight the benefits of administering low thoracic PCEA for postcesarean women who are more resistant to receiving rescue analgesics. Conversely, we observed unsatisfactory VAS profiles among participants in the other three groups at 24 h after surgery; this finding corresponds to those of previous studies [15,23,31,32,33,34,35,36]. The dermatomes of a cesarean wound are located approximately between T10 and T12 [41] and uterine cramps are also transmitted through the visceral afferent nerves entering the spinal cord from T10 through L1 [12]. Therefore, the enhanced epidural catheter–incision congruency of low thoracic PCEA alleviates postcesarean pain more efficiently than do other approaches. By contrast, lumbar epidural is incongruent to both wound pain and uterine cramp, which was emphasized in a study by Kaufner et al., who reported that lumbar PCEA was less effective than intrathecal or epidural morphine in alleviating postcesarean pain [23]. 

Intrathecal morphine is considered the gold standard single-shot drug for postcesarean pain [42]; however, its use should be balanced against the increased risk of adverse maternal effects [43]. For example, parturients are more susceptible to intrathecal opioid-induced pruritus with reported incidences of 60%–100% [26], and Asian patients are more susceptible to opioid-induced pruritus than are Caucasian patients [44]. At our institute, a high incidence of intrathecal morphine-induced pruritus (up to 80%) was also observed among parturients undergoing cesarean delivery even when a low dose (0.1 mg) was administered. By comparison, epidural morphine has been reported to be more effective than intrathecal morphine for cesarean delivery [45,46,47] while inducing less severe pruritus. Therefore, epidural morphine was the preferred neuraxial opioid at our institute. Nevertheless, we still observed that the analgesic effects of low thoracic PCEA were superior to those of epidural morphine; this may be because the common PCEA regimen included both local anesthetic and lipophilic opioid, which have greater blockage of pain pathway spread than epidural morphine has (when used alone) once the epidural is catheter–incision congruent. Moreover, the spinal effect is speculated to be the key analgesic mechanism of epidural morphine [48]. A study reported that epidural morphine administered at L4 elicited a significant analgesic effect not only at L4 at 2 h after administration but at T10 at 5 h after administration [49]. Therefore, compared with lumbar epidural morphine, an advantage of low thoracic epidural morphine may be a faster onset. However, this benefit is clinically irrelevant when a full dose of spinal anesthesia is administered during surgery. Accordingly, we observed similar analgesic profiles at 5 h after spinal anesthesia (T_1_) among participants in the low thoracic morphine and lumbar morphine groups.

In this study, we observed that the incidence and severity of pruritus and PONV were similar among the four groups. Furthermore, the participants in the four groups did not report any effects of sedation during the investigation period. However, the negative influence of epidural local anesthetic infusion on maternal motility must be considered [50]. In this study, we observed participants in the low thoracic PCEA group exhibited a Bromage motor scale profile similar to that of participants who received epidural morphine, but more participants in the lumbar PCEA group experienced prolonged lower extremity weakness after spinal anesthesia. Recently, Murata et al. reported findings related to a retrospective cohort of 205 parturients who received combined spinal-epidural anesthesia for cesarean delivery; they also discovered that epidural catheter placement at the low thoracic interspace reduced lower extremity weakness [51]. However, high heterogeneity in epidural catheter sites was observed in that study because the epidurals were inserted by performing landmark palpation, which lacks the precision required for definitive localization at the epidural level [17]. In addition, motor weakness was not subjectively defined. By contrast, ultrasound was used for epidural insertion, and a modified Bromage motor scale was used to assess lower extremity weakness in the present study. Furthermore, we also observed a significantly earlier first postoperative passage of flatulence among participants who received low thoracic PCEA. This finding corresponds to that of a previous Cochrane analysis, which reported that compared with opioid-based regimens, an epidural local anesthetic regimen led to substantially reduced postoperative gastrointestinal paralysis after abdominal surgery [52]. The technical and anatomical difficulties related to low thoracic epidural insertion are another concern. The low thoracic vertebrae are similar in spinous process angulation [53] and ultrasonographic appearance to lumbar vertebrae [54]. Therefore, a low thoracic epidural is no more difficult to perform than a lumbar epidural [55]. We observed no significant difference in total procedure time and number of epidural insertion redirections between the low thoracic and lumbar epidural groups. Therefore, this technical issue is irrelevant to our comparison of low thoracic and lumbar epidural analgesia.

This study has several limitations. First, this study was a single-center study, and institutional variations may limit the application of low thoracic PCEA for postcesarean pain control. Second, in the present study, experienced providers placed all catheters. Therefore, this study does not guarantee the generalizability of the study findings for inexperienced hands. Third, in the present study, PCEA was administered using a programmed intermittent bolus, which could spread more extensively than continuous infusion at a fixed rate [56]. Therefore, our results must be cautiously interpreted in the context of PCEA with continuous infusion. Theoretically, the differences in the analgesic effects of low thoracic and lumbar PCEA may be substantial in a continuous infusion setting, because an incongruent lumbar epidural catheter may result in less low thoracic dermatome spread. 

In conclusion, the present study verified that epidural catheter–incision congruency profoundly influenced the effects of PCEA on postcesarean pain management. A low thoracic PCEA is associated with superior analgesic efficacy relative to lumbar PCEA and epidural morphine for postcesarean pain and is without additional adverse effects or technical difficulties. Furthermore, low thoracic PCEA may promote gastrointestinal function recovery after cesarean delivery.

## Figures and Tables

**Table 1:** Participant demographics and preoperative obstetric data.

	Low Thoracic PCEA	Lumbar PCEA	Low Thoracic Morphine	Lumbar Morphine	*p* Value
	(*n* = 45)	(*n* = 49)	(*n* = 49)	(*n* = 46)	
**Age (yr)**	36.1 ± 4.5	35.0 ± 4.5	36.4 ± 4.2	36.7 ± 4.2	*p* = 0.225
**Height (cm)**	159.3 ± 6.1	159.1 ± 5.1	161.3 ± 5.5	159.5 ± 5.6	*p* = 0.204
**Weight (kg)**	69.5 ± 11.1	67.6 ± 10.3	71.9 ± 11.0	69.8 ± 10.4	*p* = 0.285
**Nulliparous (*n*; %)**	27 (60.0%)	27 (55.1%)	32 (65.3%)	27 (58.7%)	*p* = 0.779
**Indication (*n*; %)**					*p* = 0.585
**Previous uterine surgery**	14 (31.1%)	22 (44.9%)	19 (38.8%)	17 (37.0%)	
**Twin**	14 (31.1%)	6 (12.2%)	6 (12.2%)	5 (10.9%)	
**Breech**	5 (11.1%)	8 (16.3%)	11 (22.5%)	9 (19.6%)	
**Fetal abnormality**	4 (8.9%)	4 (8.2%)	3 (6.1%)	3 (6.5%)	
**Maternal request**	3 (6.7%)	4 (8.2%)	3 (6.1%)	3 (6.5%)	
**Other**	5 (11.1%)	5 (10.2%)	7 (14.3%)	9 (19.5%)	
**Preoperative questionnaire**					*p* = 0.342
**Preoperative anxiety (0–100)**	54 ± 27	62 ± 24	55 ± 25	56 ± 26	
**Anticipated pain (0–100)**	70 ± 20	76 ± 17	66 ± 19	68 ± 22	*p* = 0.081
**Anticipated medication (0–5)**	4 (3–4)	4 (3–4)	3 (3–4)	4 (3–4)	*p* = 0.208

**Table 2:** Intraoperative profiles.

	Low Thoracic PCEA	Lumbar PCEA	Low Thoracic Morphine	Lumbar Morphine	*p* Value
	(*n* = 45)	(*n* = 49)	(*n* = 49)	(*n* = 46)	
**Surgical profile**					
**Operation time (min)**	50.0 ± 14.4	50.7 ± 12.2	49.4 ± 14.2	50.0 ± 12.2	*p* = 0.977
**Blood loss (mL)**	422 ± 173	437 ± 170	431 ± 119	414 ± 89	*p*= 0.878
**Epidural profile**					
**Skin-epidural depth (cm)**	4.8 ± 0.6	4.8 ± 0.7	4.8 ± 0.8	4.9 ± 0.8	*p* = 0.755
**Procedure time (sec)**	313 ± 309	299 ± 255	272 ± 203	309 ± 341	*p* = 0.891
**Redirection (*n*)**	1.4 ± 1.3	2.0 ± 3.7	1.6 ± 1.6	1.9 ± 1.8	*p* = 0.488
**Anesthesia profile**					
**SA level (*n*; %)**					*p* = 0.054
**T6 or above**	45 (100%)	46 (93.9%)	49 (100%)	42 (91.3%)	
**Below T6**	0 (0%)	3 (6.1%)	0 (0%)	4 (8.7%)	*p* = 0.676
**Intravenous fluid (mL)**	1009 ± 345	1029 ± 281	966 ± 295	963 ± 338	
**Norepinephrine (mcg)**	25 ± 21	25 ± 21	24 ± 25	28 ± 33	*p* = 0.895

SA = spinal anesthesia.

**Table 3:** Adverse effects profiles.

	Low Thoracic PCEA	Lumbar PCEA	Low Thoracic Morphine	Lumbar Morphine	*p* Value
	(*n* = 45)	(*n* = 49)	(*n* = 49)	(*n* = 46)	
**Worst Bromage score (*n*; %)**					*p* = 0.005
**0**	41 (91.1%) ^#^	34 (69.4%) *	43 (87.7%) ^#^	42 (91.3%) ^#^	
**1**	4 (8.9%)	9 (18.4%)	4 (8.2%)	3 (6.5%)	
**2**	0 (0%)	5 (10.2%)	2 (4.1%)	1 (2.2%)	
**3**	0 (0%)	1 (2.0%)	0 (0%)	0 (0%)	
**Pruritus (*n*; %)**					*p* = 0.099
**Mild or none**	32 (71.1%)	40 (81.6%)	36 (73.5%)	33 (71.7%)	
**Moderate**	12 (26.7%)	9 (18.4%)	13 (26.5%)	9 (19.6%)	
**Severe**	1 (2.2%)	0 (0%)	0 (0%)	4 (8.7%)	
**PONV (*n*; %)**					*p* = 0.221
**None**	40 (88.9%)	40 (81.6%)	37 (75.5%)	38 (82.6%)	
**Nausea**	2 (4.4%)	8 (16.3%)	4 (8.2%)	4 (8.7%)	
**Retch**	0 (0%)	0 (0%)	1 (2.0%)	0 (0%)	
**Vomiting**	3 (6.7%)	1 (2.1%)	7 (14.3%)	4 (8.7%)	
**Ramsay Sedation Scale ≠ 2**	0 (0%)	0 (0%)	0 (0%)	0 (0%)	*p* = 0966
**First passage of flatulence (hr)**	17.3 ± 7.5	20.8 ± 8.4	26.2 ± 7.9 *^#^	25.1 ± 7.6 *^#^	*p* < 0.001
**Recovery numeric rating scale (0–100)**	76.2 ± 10.1 ^#^	70.8 ± 7.4 *	76.9 ± 8.7 ^#^	78.3 ± 8.6 ^#^	*p* < 0.001

PONV = postoperative nausea and vomiting. * means a *p* value < 0.05 compared to that of the low thoracic PCEA group. ^#^ means a *p* value < 0.05 compared to that of the lumbar PCEA group.

**Table 4:** Analgesic consumptions.

	Low Thoracic PCEA	Lumbar PCEA	Low Thoracic Morphine	Lumbar Morphine	*p* Value
	(*n* = 45)	(*n* = 49)	(*n* = 49)	(*n* = 46)	
**PCEA profile**	206 (193–228)	275 (228–308)	NA	NA	*p*< 0.001
**Total dose (mL)**					
**Demand number**	9 (4–25)	21 (9–37)			*p* = 0.007
**Delivery number**	7 (2–18)	11 (7–25)			*p* = 0.002
**Demand/delivery ratio**	1.8 ± 1.2	1.6 ± 0.9			*p* = 0.371
**Total epidural morphine dose (mg)**	NA	NA	10 (10–11.3)	11 (10–11)	*p* = 0.686
**Number of rescue intervention (*n*)**	1.0 ± 1.1 ^#^	2.5 ± 1.5 *	1.6 ± 1.8 ^#^	1.7 ± 1.7 *	*p* < 0.001
**Supplement epidural morphine dose (mg)**	0.1 ± 0.4	0.7 ± 0.9 *	0.9 ± 1.1 *	0.8 ± 1.0 *	*p* < 0.001
**IV analgesic dose**					
**Nabuphine (mg)**	1.1 ± 3.2	2.5 ± 5.2	3.0 ± 6.7	2.6 ± 7.1	*p* = 0.442
**Tenoxicam (mg)**	18.7 ± 20.2	23.7 ± 21.5	16.3 ± 19.9	21.7 ± 20.1	*p* = 0.301

PCEA = patient-controlled epidural analgesia; PCA = patient-controlled analgesia; IV = intravenous. * means a *p* value < 0.05 compared to that of the low thoracic PCEA group. ^#^ means a *p* value < 0.05 compared to that of the lumbar PCEA group. As the PCEA regimen was not used in the two morphine groups, the comparisons of the PCEA profiles were presented as non-applicable (NA) in the two morphine groups. Furthermore, the total epidural morphine doses of the two morphine groups were inevitably higher than those of the two PCEA groups; the comparison of total epidural morphine dose was not applied in the PCEA groups and was marked as NA.

## Data Availability

The data presented in this study are available on request from the corresponding author.

## Supplementary Materials

The following are available online at https://www.mdpi.com/article/10.3390/jpm11111099/s1, Table S1: Proportions of participants with a VAS of >33 mm.

## Clinical Trial Registration

The study was registered at clinicaltrials.gov with the identifier NCT03946982. Please refer to https://clinicaltrials.gov/ct2/show/NCT03946982 (accessed on 30 June 2021).
