# Supplementary material for: Influence of Catheter–Incision Congruency in Epidural Analgesia on Postcesarean Pain Management: A Single-Blinded Randomized Controlled Trial
Source: J Pers Med. 2021 Oct 27;11(11):1099. doi: 10.3390/jpm11111099 (PMC8619661; doi:10.3390/jpm11111099)
Supplement: Supplementary file 1 [file jpm-11-01099-s001.zip › jpm-1427342-supplementary.pdf]

Supplemental Digital Content

Table S1. Proportions of participants with a VAS of >33 mm.

	Low thoracic PCEA (N= 45)	Lumbar PCEA (N= 49)	Low thoracic morphine (N= 49)	Lumbar morphine (N= 46)	p value
T_1 (n; %)					
Static pain					
VAS = 0	12 (26.7%)#	2 (4.1%)*	4 (8.2%)*	0 (0%)*	p< 0.001
VAS> 33 mm	7 (15.6%)#	23 (46.9%)*	18 (36.7%)*	25 (54.3%)*	p< 0.001
Dynamic pain					
VAS = 0	7 (15.6%)#	2 (4.1%)*	3 (6.1%)	0 (0%)*	p = 0.019
VAS> 3.3	13 (28.8%)#	34 (69.4%)*	33 (67.3%)*	34 (73.9%)*	p < 0.001
Uterine cramp					
VAS = 0	10 (22.2%)#	0 (0%)*	0 (0%)*	3 (6.5%)*	p < 0.001
VAS> 33 mm	18 (40.0%)#	30 (61.2%)*	37 (75.5%)*	32 (69.6%)*	p = 0.003
T_2 (n; %)					
Static VAS					
VAS = 0	7 (15.6%)	3 (6.1%)	2 (4.1%)	2 (4.3%)	p = 0.119
VAS> 33 mm	4 (8.9%)	13 (26.5%)	12 (24.5%)	10 (21.7%)	p = 0.152
Dynamic VAS					
VAS = 0	2 (4.4%)	0 (0%)	0 (0%)	0 (0%)	p = 0.092
VAS> 33 mm	16 (35.6%)#	29 (59.2%)*	30 (61.2%)*	28 (60.7%)*	p = 0.036
Uterine cramp VAS					
VAS = 0	3 (6.7%)	0 (0%)	0 (0%)	1 (2.2%)	p = 0.086
VAS> 33 mm	18 (40.0%)#	32 (65.3%)*	22 (44.9%)#	16 (34.8%)#	p = 0.016
T_3 (N; %)					
Static pain					
VAS = 0	10 (22.2%)#	1 (2.0%)*	5 (10.2%)*	3 (6.5%)*	p = 0.010
VAS> 33 mm	0 (0%)	6 (12.2%)	6 (12.2%)	2 (4.3%)	p = 0.057
Dynamic pain					
VAS = 0	1 (2.2%)	0 (0%)	0 (0%)	1 (2.2%)	p = 0.539
VAS> 33 mm	9 (20.0%)#	26 (57.8)*	19 (38.8%)	22 (47.8%)*	p = 0.007
Uterine cramp					
VAS = 0	4 (8.9%)	1 (2.0%)	0 (0%)	2 (4.3%)	p = 0.128
VAS> 33 mm	9 (20.0%)#	22 (44.9%)*	12 (24.5%)#	10 (21.7%)#	p = 0.023
T_4 (N; %)					
Static pain					
VAS = 0	12 (26.7%)	5 (10.2%)	12 (24.5%)	9 (19.6%)	p = 0.188
VAS> 33 mm	1 (2.2%)	4 (8.2%)	3 (6.1%)	1 (2.2%)	p = 0.431
Dynamic pain					
VAS = 0	1 (2.2%)	1 (2.0%)	1 (2.0%)	1 (2.2%)	p = 0.999
VAS> 33 mm	9 (20.0%)&	13 (26.5%)	20 (40.8%)*	8 (17.4%)&	p = 0.045
Uterine cramp					
VAS = 0	3 (6.7%)	2 (4.1%)	5 (10.2%)	3 (6.5%)	p = 0.692
VAS> 33 mm	10 (22.2%)	10 (20.4%)	10 (20.4%)	2 (4.3%)	p = 0.076

* means a p value< 0.05 comparing to that of low thoracic PCEA group. # means a p value< 0.05 comparing to that of lumbar PCEA group. & means a p value< 0.05 comparing to that of low thoracic morphine group.
